# Etiology of pulmonary venous aneurysm diagnosed by a combination of echocardiography and contrast-enhanced computed tomography: a case report

**DOI:** 10.1186/s13019-014-0132-6

**Published:** 2014-09-20

**Authors:** Yun Mou, Yan Cheng, Qiang Feng, Chenyao Ni

**Affiliations:** Department of Ultrasound, The First Affiliated Hospital, College of Medicine, Zhejiang University, #79 Qingchun Road, Hangzhou, China; Department of Cardiothoracic Surgery, The First Affiliated Hospital, College of Medicine, Zhejiang University, #79 Qingchun Road, Hangzhou, China

**Keywords:** Pulmonary venous aneurysm, Mitral regurgitation, Contrast-enhanced computed tomography, Echocardiography

## Abstract

Pulmonary venous aneurysm is rare and its etiology is still debated. Some studies have shown that acquired aneurysms are caused by an increase in left atrial pressure and mitral regurgitation. However, few reports have provided direct evidence of this pathology. We present a case of pulmonary venous aneurysm diagnosed using a combination of echocardiography and contrast-enhanced computed tomography with strong evidence for an etiology of severe mitral valve regurgitation. A 24-year-old Chinese man was diagnosed with mitral valve perforation with severe mitral regurgitation caused by infectious endocarditis. A right inferior pulmonary venous aneurysm was diagnosed by contrast-enhanced computed tomography. Color Doppler echocardiography revealed a mitral regurgitation jet filling the giant aneurysm. Three-dimensional echocardiography provided a clear stereoscopic view of the aneurysm. These imaging modalities provided direct evidence for the etiology of the pulmonary venous aneurysm.

**Electronic supplementary material:**

The online version of this article (doi:10.1186/s13019-014-0132-6) contains supplementary material, which is available to authorized users.

## Background

Pulmonary venous aneurysm (PVA) is rare, often presenting as a mediastinal mass [1]-[3], with arteriovenous malformation being the most common cause. More than 80% of cases are congenital, of which half or more are associated with hereditary hemorrhagic telangiectasia [4]. Some studies have shown that acquired aneurysms are the result of an increase in left atrial pressure and mitral regurgitation [1]-[3]. However, few reports have provided direct evidence of this pathology. We present a case of PVA with strong evidence for an etiology of severe mitral valve regurgitation.

## Case presentation

The study was approved by the Institutional Review Board at the First Affiliated Hospital, College of Medicine, Zhejiang University. The procedures were conducted according to the principles of the Helsinki Declaration.

**Figure 1:**
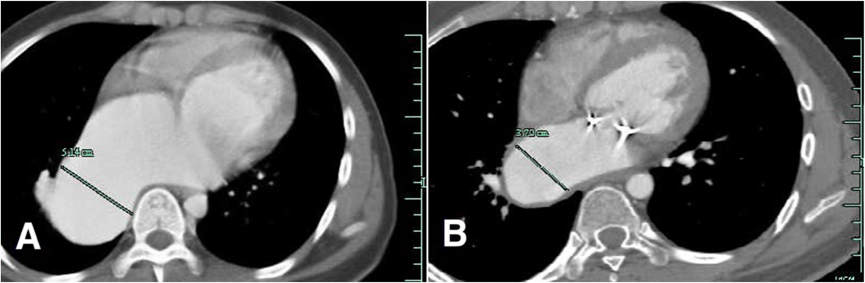
**Comparison of the right inferior PVA pre and postoperatively. (A)** The PVA is 5.14 cm in diameter preoperatively. **(B)** Three months postoperatively, the PVA is decreased in size. PVA, pulmonary venous aneurysm.

**Figure 2:**
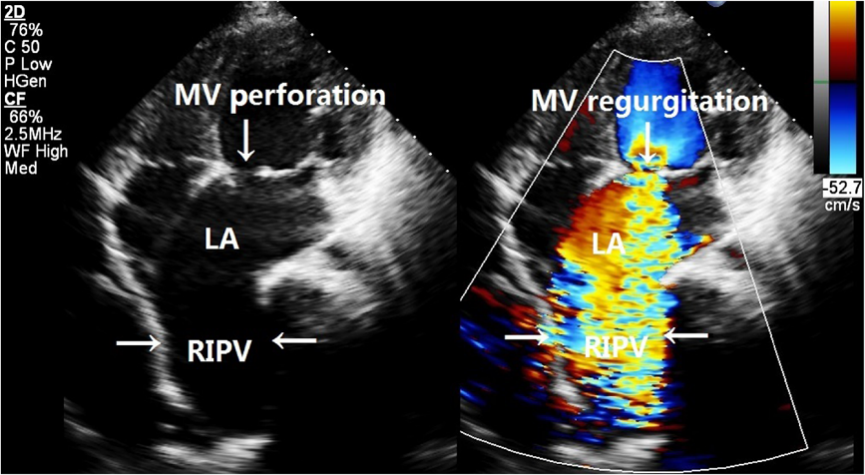
**Echocardiographic images in split-screen mode revealing two-dimensional echocardiography and color Doppler simultaneously.** Images showing high-velocity turbulent jet caused by perforation of the mitral valve filling in the RIPV. LA, left atrium; MV, mitral valve; RIPV, right inferior pulmonary vein.

**Figure 3:**
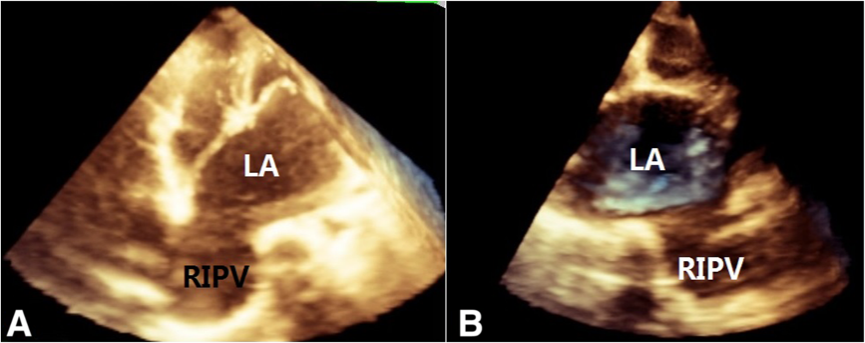
**Three-dimensional echocardiography showing a clear stereoscopic view of the right pulmonary venous aneurysm. (A)** The three-dimensional echocardiographic view in apical view reveals the enlarged LA and RIPV. **(B)** The three-dimensional echocardiographic view in parasternal view shows the large LA and RIPV. LA, left atrium; RIPV, right inferior pulmonary vein.

A 24-year-old Chinese man was referred to our hospital because of chest tightness and dyspnea due to progressive heart failure. Fifteen years earlier, he had been diagnosed with *Staphylococcus aureus* sepsis and infectious endocarditis with mitral valve vegetations and insufficiency. Chest x-ray was normal at that time and three years prior to presentation. On presentation, physical examination revealed a grade 4 holosystolic murmur heard best at the left sternal border and apex, mild lower extremity edema, and no hepatomegaly, ascites. Echocardiography showed a significantly enlarged left atrium (4.7 cm in diameter) and left ventricle (7.6 cm in diameter). Severe mitral valve regurgitation was caused by an approximately 1 cm sized perforation in the A3 segment of the anterior leaflet. Electrocardiogram revealed atrial fibrillation. Chest x-ray showed a mass in the mediastinum adjacent to the right border of the heart, and contrast-enhanced chest computed tomography (CT) revealed the mass to be a right inferior PVA (Figure 1). To study the case more carefully, we performed repeat echocardiography, which revealed severe mitral regurgitation with a turbulent high-velocity jet extending to a giant right inferior pulmonary vein during systole (Figure 2). Three-dimensional echocardiography enabled a stereoscopic view of the right PVA (Figure 3). The patient underwent mitral valvuloplasty, but no surgical intervention for the aneurysm was performed. Transesophageal echocardiography showed mild mitral regurgitation remained. The patient underwent contrast-enhanced CT and echocardiography 3 months postoperatively, which showed that the aneurysm had decreased in size (Figure 1).

The etiology of PVA is still debated [2],[4]-[6]. DeBoer et al. performed pulmonary lobectomy and excision of the corresponding pulmonary venous system for surgical correction of a PVA [2]. The pathological findings were nonspecific. These included focal interruptions in the elastic lamina and areas of complete absence of elastic lamina. But the etiology of the PVA was unclear. In 2004, Beckert and Jander reported a case of PVA in which transesophageal echocardiography revealed severe mitral regurgitation directed toward a dilated pulmonary vein; the PVA completely disappeared 6 months after mitral valve repair [6]. In a report by Erkanli et al. [4], atriotomy revealed a left inferior PVA with an enlarged left atrium, with the aneurysm located opposite the mitral valve. After ring valvuloplasty of the mitral valve, the left atrium decreased in size. The authors speculated that the PVA was secondary to mitral regurgitation.

We report a case of PVA diagnosed using a combination of echocardiography and contrast-enhanced chest CT, which provided strong evidence for an etiology of severe mitral regurgitation. In this case, we excluded the possibility of congenital PVA on the basis of previous x-ray examinations. Echocardiography revealed that the mitral regurgitation jet flowed toward the main right inferior pulmonary vein. The vein was filled with high-velocity turbulence downstream and was much larger than the right superior pulmonary vein, left inferior pulmonary vein, and left superior pulmonary vein. The latter three veins were not reached by the jet. Therefore, it was reasonable to presume that the main cause of the right inferior PVA was mitral regurgitation rather than enlargement of the left atrium and increased left atrial pressure. The patient's 3-month follow-up results confirmed our hypothesis.

Color Doppler echocardiography was helpful in the diagnosis. However, initially we missed the diagnosis, incorrectly considering the giant inferior right pulmonary vein to be part of left atrium because of the significant decrease in pulmonary venous flow velocity with increased left atrial pressure. This made observation of venous flow difficult on transthoracic echocardiography [7]. We performed transthoracic echocardiography a second time, after obtaining a CT scan with a clear stereoscopic view of the aneurysm provided by three-dimensional echocardiography, which was also helpful in diagnosing the PVA.

We also believe contrast-enhanced chest CT is important in the diagnosis of PVA. Although a CT with contrast cannot show the dynamic situation of the heart and vessels, it can give a precise anatomic view of the pulmonary veins. In this case, it revealed the mediastinal mass to be a giant pulmonary vein and eliminated the suspicion of arteriovenous malformation in the lung, although it could not determine the etiology of the disease. Venography is the diagnostic gold standard for PVA, but it may lead to thrombosis and result in serious complications [8]. We found the combination of echocardiography and contrast CT to facilitate accurate and safe diagnosis of PVA.

## Conclusions

The combination of contrast-enhanced CT scan and echocardiography facilitated the diagnosis of PVA. Acquired PVA may result from severe mitral value regurgitation. This etiology can be revealed by color Doppler echocardiography, which can clearly show the relationship of the regurgitation jet and the PVA.

## Consent

Written informed consent was obtained from the patient for publication of this case report and the accompanying images. A copy of the written consent is available for review by the editor-in-chief of this journal.

## Authors’ original submitted files for images

Below are the links to the authors’ original submitted files for images. Authors’ original file for figure 1 Authors’ original file for figure 2 Authors’ original file for figure 3

## Abbreviations

PVA: Pulmonary venous aneurysm
CT: Computed tomography
LA: Left atrium
MV: Mitral valve
RIPV: Right inferior pulmonary vein

## References

[1] Sirivella S, Gielchinsky I (1999). Pulmonary venous aneurysm presenting as a mediastinal mass in ischemic cardiomyopathy. Ann Thorac Surg.

[2] DeBoer DA, Margolis ML, Livornese D, Bell KA, Livolsi VA, Bavaria JE (1996). Pulmonary venous aneurysm presenting as a middle mediastinal mass. Ann Thorac Surg.

[3] Liao WN, Huang CC, Huang JK, Shih SL (2012). Pulmonary venous aneurysm mimicking a right infrahilar tumour. Eur J Cardiothorac Surg.

[4] Erkanli K, Yazici P, Bakir I (2014). Pulmonary vein aneurysm secondary to mitral regurgitation: rare and confusing lesion. Thorac Cardiovasc Surg.

[5] Restrepo CS, Carswell AP (2012). Aneurysms and pseudoaneurysms of the pulmonary vasculature. Semin Ultrasound CT MR.

[6] Beckert J, Jander N (2004). Complete regression of pulmonary vein aneurysm caused by mitral regurgitation. Heart.

[7] Tabata T, Thomas JD, Klein AL (2003). Pulmonary venous flow by Doppler echocardiography: revisited 12 years later. J Am Coll Cardiol.

[8] de Bono D (1993). Complications of diagnostic cardiac catheterisation: results from 34,041 patients in the United Kingdom confidential enquiry into cardiac catheter complications. Br Heart J.

