# Supplementary figures and images for: Etiology of pulmonary venous aneurysm diagnosed by a combination of echocardiography and contrast-enhanced computed tomography: a case report
Source: J Cardiothorac Surg. 2014 Sep 20;9:132. doi: 10.1186/s13019-014-0132-6 (PMC4172824; doi:10.1186/s13019-014-0132-6)

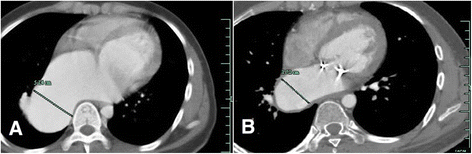

Supplement: Supplementary file 1 — Authors’ original file for figure 1 [file 13019_2014_132_MOESM1_ESM.gif]

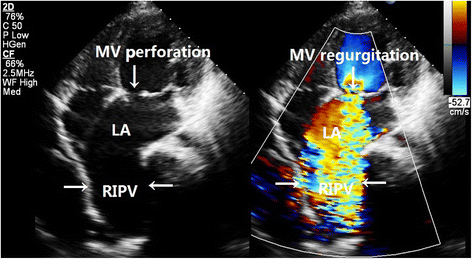

Supplement: Supplementary file 2 — Authors’ original file for figure 2 [file 13019_2014_132_MOESM2_ESM.gif]

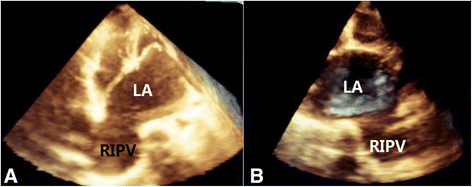

Supplement: Supplementary file 3 — Authors’ original file for figure 3 [file 13019_2014_132_MOESM3_ESM.gif]
